# Mitochondrial impairment and synaptic dysfunction are associated with neurological defects in iPSCs-derived cortical neurons of MERRF patients

**DOI:** 10.1186/s12929-023-00966-8

**Published:** 2023-08-21

**Authors:** Yu-Ting Wu, Hui-Yi Tay, Jung-Tse Yang, Hsiao-Hui Liao, Yi-Shing Ma, Yau-Huei Wei

**Affiliations:** 1 Center for Mitochondrial Medicine and Free Radical Research, Changhua Christian Hospital, Changhua City, Taiwan 50046; 2 Institute of Clinical Medicine, National Yang Ming Chiao Tung University, Taipei, Taiwan 112

**Keywords:** AMPARs, Disease modeling, Electrophysiological activity, Excitatory neurons, iPSCs, MERRF syndrome, mtDNA mutation, Neurological defect, Synaptic plasticity, Synaptophysin

## Abstract

**Background:**

Myoclonic epilepsy with ragged-red fibers (MERRF) syndrome is a rare inherited mitochondrial disease mainly caused by the m.8344A > G mutation in mitochondrial tRNA^Lys^ gene, and usually manifested as complex neurological disorders and muscle weakness. Currently, the pathogenic mechanism of this disease has not yet been resolved, and there is no effective therapy for MERRF syndrome. In this study, MERRF patients-derived iPSCs were used to model patient-specific neurons for investigation of the pathogenic mechanism of neurological disorders in mitochondrial disease.

**Methods:**

MERRF patient-derived iPSCs were differentiated into excitatory glutamatergic neurons to unravel the effects of the m.8344A > G mutation on mitochondrial bioenergetic function, neural-lineage differentiation and neuronal function. By the well-established differentiation protocol and electrophysiological activity assay platform, we examined the pathophysiological behaviors in cortical neurons of MERRF patients.

**Results:**

We have successfully established the iPSCs-derived neural progenitor cells and cortical-like neurons of patients with MERRF syndrome that retained the heteroplasmy of the m.8344A > G mutation from the patients’ skin fibroblasts and exhibited the phenotype of the mitochondrial disease. MERRF neural cells harboring the m.8344A > G mutation exhibited impaired mitochondrial bioenergetic function, elevated ROS levels and imbalanced expression of antioxidant enzymes. Our findings indicate that neural immaturity and synaptic protein loss led to the impairment of neuronal activity and plasticity in MERRF neurons harboring the m.8344A > G mutation. By electrophysiological recordings, we monitored the in vivo neuronal behaviors of MERRF neurons and found that neurons harboring a high level of the m.8344A > G mutation exhibited impairment of the spontaneous and evoked potential-stimulated neuronal activities.

**Conclusions:**

We demonstrated for the first time the link of mitochondrial impairment and synaptic dysfunction to neurological defects through impeding synaptic plasticity in excitatory neurons derived from iPSCs of MERRF patients harboring the m.8344A > G mutation. This study has provided new insight into the pathogenic mechanism of the tRNA^Lys^ gene mutation of mtDNA, which is useful for the development of a patient-specific iPSCs platform for disease modeling and screening of new drugs to treat patients with MERRF syndrome.

**Supplementary Information:**

The online version contains supplementary material available at 10.1186/s12929-023-00966-8.

## Background

Mitochondria carry out numerous biochemical reactions in energy metabolism and biosynthesis of pyrimidine nucleotides and steroid hormones and play a vital role in the regulation of cell fate. Aside from the bioenergetic function, mitochondria are involved in the regulation of redox status, Ca^2+^ homeostasis, adaptive response and apoptosis. Mitochondria form network structures with highly dynamic activity in response to energy demand and facilitate cells to cope with the environmental and physiological changes in coordination with other intracellular organelles. It has been suggested that mitochondrial dysfunction is linked to neurodegenerative diseases, aging, cancer, and metabolic diseases.

The MERRF syndrome, also known as myoclonic epilepsy with ragged red fibers, is a rare mitochondrial disease that mainly affects the nervous system and muscles. The m.8344A > G mutation in the mitochondrial tRNA^Lys^ gene has been identified as the major primary genetic defect causing the MERRF syndrome, which hinders protein synthesis in mitochondria and leads to bioenergetic dysfunction [1]. Mitochondrial genome (mtDNA) presents multiple copy in cells and contains 37 genes that encode 13 polypeptides, 22 tRNAs, and 2 rRNAs. The coordination between mtDNA-encoded polypeptides with nuclear DNA-encoded proteins is essential for the structural integrity and function of mitochondria. Specific gene mutations that occur in the nuclear DNA or mtDNA can impair the subcomplexes and regulatory factors of electron transport chain and results in oxidative phosphorylation (OXPHOS) deficiency [2]. Since mtDNA is vulnerable to ROS attack, its mutations accumulate much faster than does the nuclear DNA. Disease-causing mtDNA mutations are maternally-inherited and coexist with the wild-type mtDNA in the affected tissues, which is termed heteroplasmy. More than 300 mutations have been identified in mtDNA that are associated with mitochondrial diseases, which are mostly presented with defects in respiration and overproduction of reactive oxygen species (ROS) [3].

MERRF syndrome has variable clinical features, making it a challenge for the diagnosis and management of the disease. Patients with the MERRF syndrome commonly exhibit progressive myoclonic epilepsy of variable severity and is often associated with a range of neurological symptoms, including myoclonus, seizures, ataxia, dementia, and neuropathy [4]. It has been shown that mitochondria play a crucial role in neuronal development, adult neurogenesis and mature neuron function [5]. In the nervous system, functional mitochondria are involved in axonal and dendritic development, axonal regeneration, and synaptic plasticity. The human brain is a highly complex organ with remarkable energy demand that accounts for about 20% of the total energy expenditure of the body. Due to a higher metabolic requirement and the limitation of glycolytic capacity, neurons depend on mitochondria to generate ATP for neurotransmission and normal electrical activity. Such metabolic characteristics make neurons vulnerable to electron leak from electron transport chain, which may cause ROS overproduction and oxidative damage. Therefore, function and survival of neurons are very sensitive to mitochondrial dysfunction. Although it has been established that pathogenic mtDNA mutations can impair the bioenergetic function of mitochondria, the underlying mechanism of mtDNA mutation in the onset and progression of neurological disorders in mitochondrial diseases remain poorly understood.

Most research has focused on how the disease-associated mtDNA mutations affect mitochondrial function and on the development of therapeutic strategies to improve mitochondrial function and ameliorate symptoms of MERRF patients [2]. However, the molecular mechanism underlying the pathogenesis of mitochondrial diseases caused by mtDNA mutations has not been fully elucidated. Since Prof. Yamanaka successfully reprogrammed adult cells into induced pluripotent stem cells (iPSCs) [6], the development of iPSCs technologies for human disease modeling has provided a new approach for pathophysiological study, drug discovery and regenerative medicine, especially in neurodegenerative and cardiovascular diseases [7]. iPSCs are the artificial stem cell populations with pluripotency and can differentiate to all cell types to offer an inexhaustible cellular resource. Several studies have used iPSCs to investigate the mechanism of pathogenesis of mitochondrial diseases, such as LHON and the MELAS syndrome [8–11]. In the past few years, we have generated several iPSCs lines by using the primary culture of skin fibroblasts from patients with MERRF syndrome and other mitochondrial diseases, respectively [12–14]. We have successfully differentiated MERRF iPSCs into inner ear hair cell-like cells [15] and cardiomyocytes [12], respectively, and both of which displayed the phenotype caused by mitochondrial dysfunction. These iPSCs-derived cells exhibited biochemical defects that have been observed in the skin fibroblasts and cybrids of patients with MERRF syndrome. However, to the best of our knowledge there is no report on the neurophysiological behaviors of iPSCs-derived neurons of MERRF syndrome. In this study, iPSCs derived from the skin fibroblasts of patients with MERRF syndrome were differentiated into neural cell lineage to model neurological disorders that are often manifested in patients with the disease. By a well-established differentiation protocol, we generated patient-specific cortical-like neurons to explore the pathogenic mechanism underlying the onset and progression of neurological disorders in patients with MERRF syndrome.

## Methods

### Cell culture of MERRF iPSCs

The 6 lines of human iPSCs used in this study were generated from the skin fibroblasts established from 3 patients harboring the MERRF syndrome-specific m.8344A > G mutation. Among them, two patients are the members of a Taiwanese MERRF family, a 15-year-old girl (M1-iPSCs) and her 13-year-old sister (M2-iPSCs), and the third patient is a 25-year-old female (M3-iPSCs) as described previously [12, 14, 16]. M1 patient had poor learning ability in childhood and developed myoclonic epilepsy at 12 years of age, and exhibited severe clinical symptoms, including unsteady gait with tremor, intermittent myoclonus and polyneuropathy [17]. Her early asymptomatic sister M2 had a later onset after tissue cells had been collected despite carrying the m.8344A > G mutation. M3 patient was diagnosed with progressive ataxia and myoclonic epilepsy at 13 years of age [18]. She had suffered from progressive weakness and atrophy of extremities for six to seven years until the tissue biopsies were obtained. The primary cultures of skin fibroblasts were established in our laboratory before 1995 from these patients who had been diagnosed to have MERRF syndrome by Dr. Yuh-Jyh Jong at the Department of Pediatrics, Kaohsiung Medical University Hospital, Kaohsiung and by Dr. Chin-Chang Huang at the Department of Neurology, Chang Gung Memorial Hospital, Lin-Kou, respectively [17, 18]. The clinical specimens of the patients were provided by the clinicians according to the guidelines set by each of the hospitals. Among all iPSCs lines, M1-iPSC sublines (M1^Low^ and M1^High^ iPSCs) and M2-iPSCs (M2^High^ iPSCs) were generated at Taipei Veterans General Hospital and have been used for the studies reported previously [12]. M3-iPSCs sublines (M3^Low^ and M3^Med^ iPSCs) were generated from another MERRF patient in collaboration with Prof. Patrick C. H. Hsieh at the Institute of Biomedical Sciences, Academia Sinica supported by Taiwan Human Disease iPSC Service Consortium [14]. Prof. Y. C. Hsu at Mackay Medical College kindly provided us the normal iPSCs (IBMS-iPSC-02-07), which were generated from the peripheral blood of a healthy 22-year-old Asian male volunteer and are commercially available from the Food Industry Research and Development Institute, Hsinchu, Taiwan. The generation of iPSCs from skin fibroblasts of MERRF patients was approved by the Institutional Review Board (IRB) of Mackay Memorial Hospital, Mackay Medical College, and Taipei Veterans General Hospital, respectively. For this study, the IRB waived the informed consent requirement for the use of these iPSCs cell lines. The ability of the MERRF iPSCs to differentiate into three germ layers in vitro and form teratoma and three germ layers in vivo was confirmed during characterization of iPSCs [12, 14]. The iPSCs were expanded in Gibco™ StemFlex™ medium on Geltrex-coated plates at 37 °C in a humidified CO_2_ incubator. For the first 24 h after seeding, the growth medium was supplemented with 10 μM Rho-associated protein kinase (ROCK) inhibitor Y27632 (Adooq, Irvine, CA, USA).

### Total DNA extraction

Total cellular DNA was isolated by phenol/chloroform extraction. The cell pellet was re-suspended in the TE buffer (10 mM Tris–HCl, 1 mM EDTA, pH 8.3) containing 1% SDS, 0.25 mg/ml RNase A and 0.4 mg/ml proteinase K and incubated at 56 °C overnight. After repeated extraction with phenol/chloroform, DNA was precipitated with ice-cold 75% ethanol, air-dried, and dissolved in distilled water for further analysis.

### Quantitative analysis of the m.8344A > G mutation by PCR–RFLP

The proportion of mtDNA with the m.8344A > G mutation in cultured cells was determined by PCR–RFLP using a forward primer L8150 (8150–8169): 5′-CCGGGGGTATACTACGGTCA-3′ and a mismatched reverse primer MR28 (8372–8345): 5′-GGGGCATTTCACTGTAAAGAGGTGCCGG-3′ as previously described [17]. The primers were designed to create a *Nae* I restriction site after PCR amplification of the DNA encompassing the putative m.8344A > G mutation in the tissue or cultured cells from MERRF patients. The 223-bp PCR product could be cleaved by *Nae* I into 197-bp and 26-bp fragments.

### Whole genome sequencing of mtDNA by NGS

Whole genome sequencing of mtDNA was performed by collaboration with The Union Clinical Laboratory (Taipei, Taiwan) using the MiSeq Next-Generation Sequencing Systems. Briefly, the full-length mtDNA was amplified by a long-range PCR method with minor modifications [19]. The PCR was performed using Platinum SuperFi II DNA polymerase (Invitrogen, Carlsbad, CA, USA) with 50 ng of genomic DNA as the template, and the thermal cycler conditions include 30 cycles of 95 °C for 20 s and 65 °C for 8 min. PCR products (16.6 kb fragments) were cleaned-up by using the Beckman Coulter™ Agencourt AMPure XP (Beckman, Brea, CA, USA). Approximately 100 ng of PCR products were sheared to approximately 120 base pairs using the M220 Focused-ultrasonicator, and library construction and sequencing were then carried out using the KAPA HyperPrep Kit and MiSeq Reagent Kit v2 (Agilent Technologies, Santa Clara, CA, USA). The mtDNA variant calling and annotation were finally performed by the mitochondrial genome mapping (rCRS, GenBank: J01415.2) [20] and databases annotation was carried out according to the manufacturer’s instructions.

### Immunofluorescence staining

Cells were fixed with 4% PFA, permeabilized and blocked with 0.3% Triton X-100, 6% bovine serum in the TBS buffer. Samples were incubated overnight at 4ºC with the primary antibodies against SEEA4 (Thermo, MA1-021-D488, 1:100), SOX2 (Thermo, MA1-014-D488, 1:100); Nestin (BioLegend, 656812, 1:100); SOX1 (Abcam, ab8775, 1:200); MAP2 (Merck Millipore, AB5622, 1:200), and TUJ1 (Abcam, ab224978, 1:200), respectively. Alexa-Fluor-Dye secondary antibodies were applied (1:500) at room temperature for 1 h. After staining, cells were washed twice and dried. Stained cells were photographed and images were acquired under a microscope of Tissue FAXS i4 SCAN (Zeiss, Dublin, CA, USA).

### Western blot analysis

The cells were lysed with lysis buffer B (50 mM HEPES, 4 mM EDTA, 2 mM EGTA, 1% Triton X-100) containing a protease inhibitor cocktail (Bioman, Taipei, Taiwan). An aliquot of 30 μg proteins were separated on 10% SDS-PAGE and blotted onto a piece of the PVDF membrane (Pall Corporation, Port Washington, NY, USA). After blocking with skim milk, the membrane was incubated with primary antibodies and then reacted with horseradish peroxidase conjugated with anti-rabbit IgG and anti-mouse IgG, respectively. The protein bands were visualized on an imaging system (Gel Doc XR^+^ system, Bio-Rad Laboratories, Inc., Hercules, CA, USA) by using an ECL chemiluminescence reagent according to the manufacturer’s instruction and each protein signal was quantified by an Image Lab software (Bio-Rad Laboratories, Inc., Hercules, CA, USA).

### Measurement of mitochondrial respiration

The Seahorse XF^e^24 Extracellular Flux Analyzer (Agilent Technologies, Santa Clara, CA, USA) was used to measure the OCR of cells in real-time at 37 °C with sequential injection of 1 μM oligomycin A (OA), 2 μM FCCP, and 2.5 μM antimycin A plus 2 μM rotenone (AA/Rot), respectively, to examine the effect of these compounds on respiration. Oligomycin A was injected to assay the ATP-coupled mitochondrial respiration rate. Injection of FCCP provided maximal mitochondrial respiration. Finally, antimycin A and rotenone were injected to estimate non-mitochondrial respiration rate. The maximal respiratory rate was calculated as the difference between the OCR after FCCP injection and the lowest OCR was reached after AA/Rot addition. OA-sensitive (ATP-coupled) respiration rate was calculated as the difference between the basal OCR and the OCR after OA injection.

### Determination of the intracellular ROS level

Intracellular H_2_O_2_ levels was measured by staining cells with 20 μM 2,7-dichlorofluorescin diacetate (DCFH-DA) (Thermo Fisher Scientific, Waltham, MA, USA), respectively, at 37°C for 20 min. Cells were resuspended in 50 mM HEPES buffer (pH 7.4) and subjected to analysis on a flow cytometer (FACSAria III, BD Biosciences, San Jose, CA, USA). The intensity of emitted fluorescence at 525 nm was recorded for DCF.

### Cortical neuronal differentiation of iPSCs

Differentiation of iPSCs to mature cortical-like excitatory neurons was performed by the two-step neural differentiation method. Combination of the small-molecule inhibitors of SMAD and GSK-3β with commonly used neural supplements can drive rapid production of neural stem cells or progenitor cells (iNSCs/iNPCs) and terminal differentiation of neurons, respectively, from iPSCs. Direct induction of iPSCs to expandable iNSCs was achieved by using the neural induction medium (Merck Millipore, Burlington, MA, USA) according to the manufacture’s protocol with modifications. On day 10 of neural induction, iNSCs were harvested and expanded. Cells were reseeded in StemPro NSC serum-free expansion medium (Gibco, Carlsbad, CA, USA) consisting of neurobasal medium supplemented with B27 supplement of bFGF, and l-glutamine. Cells were either expanded for banking or used for neuron differentiation between 3 and 8 passages. For terminal neuron differentiation, iNSCs were plated on poly-l-ornithine (Sigma Aldrich Chemical Co., St. Louis, MO, USA) and laminin (Gibco, Carlsbad, CA, USA) at 1–2 × 10^4^ cells/cm^2^ with the differentiation medium (Merck Millipore, Burlington, MA, USA) supplemented with 0.5 mM dibutyryl cAMP and 0.2 mM ascorbic acid phosphate. Differentiation medium was refreshed every 2 days for the duration of 7–70 days. Mature neurons were harvested for characterization of protein markers by immunofluorescence staining and Western blot analysis of the protein expression levels of neuron-specific genes.

### Measurement of electrophysiological activity and electrical stimulation

Human iPSC-derived iNSCs were seeded at 6 × 10^4^ cells in a 24-well microelectrode array (MEA) plate (Axion Biosystems, Atlanta, GA, USA) containing 16 electrodes for the assay of neuron differentiation and maturation. All procedures were conducted by following the user’s manual of Axion Biosystems. MEAs system can capture the extracellular field potentials of electrically active cells by recording their neuronal activity [21]. To examine the spontaneous electrical activity of differentiated neuron, we collected the activity data from neuronal networks for 8 min by MEA every two or three days starting from day 14 after neuronal differentiation (3–4 replicates per experiment). Spike and burst rates represented the overall activity of the neuronal network, with more spikes and bursts corresponding to a higher electric activity. Network events include synchronous network spikes and bursts in which more than 35% electrodes captured the activity simultaneously. For electrical stimulation, after differentiation for 8 weeks the neurons received current injection at 50 μA/1000 μs/1000 mV every 5 s for five consecutive stimulations.

### Statistical analysis

Statistical analysis was performed by the Microsoft Excel 2013 statistical package and the data are presented as means ± SEM of the results obtained from 3 independent experiments. The significance level of the difference between control and the experimental groups was determined by Student's *t* test. A difference is considered significant when *p value < 0.05, **p value < 0.01, and ***p value < 0.001.

### Linear regression analysis

Linear regression analysis was performed by the Microsoft Excel to estimate the correlation between the m.8344A > G mutation load and biological function data. Regression output values including the coefficients, R-squared values and p-values are summarized in the Additional file 1: Table S1.

## Results

### Effect of the m.8344A > G mutation on mitochondrial bioenergetic function of MERRF-iPSCs

**Fig. 1:**
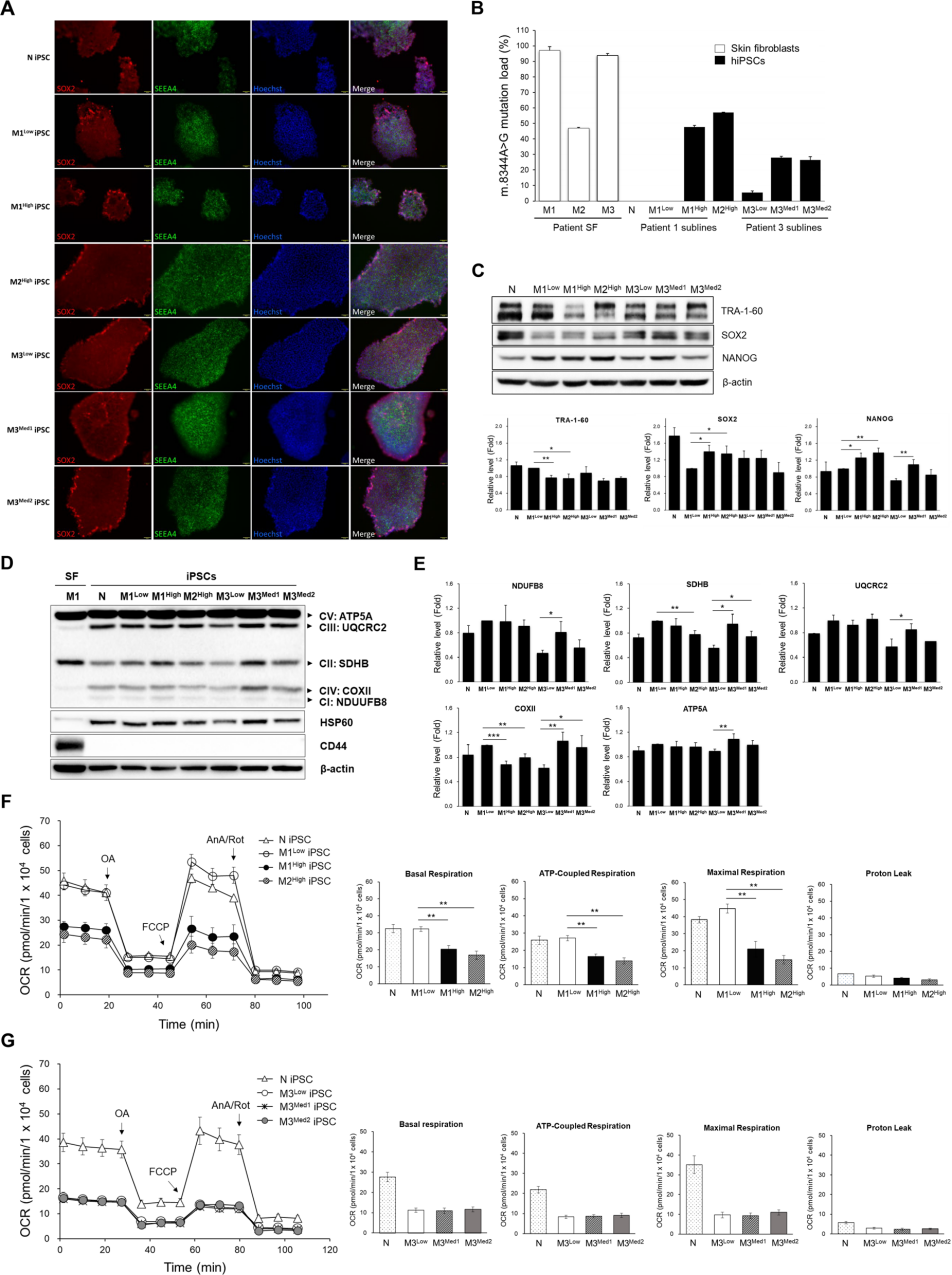
Effect of the m.8344A > G mutation on mitochondrial bioenergetic function of MERRF-iPSCs. **A** Characterization of all MERRF-iPSCs lines by the immunofluorescence staining with pluripotency markers, SOX2 (red), SEEA4 (green) and Hoechst 33342 (blue). Scale bars, 100 μm. **B** Preservation of the heteroplasmy levels of the m.8344A > G mutation in MERRF-iPSCs. The proportion of mtDNA with the m.8344A > G mutation was quantified by PCR–RFLP in the skin fibroblasts from patients with MERRF syndrome, normal (N) and MERRF-iPSCs sublines. **C** The protein expression patterns and quantification of stemness genes in normal and MERRF-iPSCs lines analyzed by Western blots. β-actin was used as the internal control. **D** The protein levels of some subunits of respiratory enzyme complexes in MERRF-iPSCs were analyzed by Western blot. **E** Comparison of the OXHPOS protein expression levels in different MERRF-iPSCs. Quantification of the proteins in Western blots was normalized with β-actin. All the data are expressed as the fold change of that of control (M1^Low^ iPSCs). **F** The representative data showing the oxygen consumption rate (OCR) in normal (N), M1^Low^, M1^High^ and M2^High^ iPSCs lines. OCR was analyzed by a Seahorse XF^e^24 extracellular flux analyzer after sequential injection of 1 μM oligomycin A (OA), 0.5 μM FCCP, and 2.5 μM antimycin A plus 2 μM rotenone (AA/Rot), respectively. Quantitative data of the bioenergetic parameters including the basal respiration rate, maximal, and ATP-coupled mitochondrial respiration rates of MERRF-iPSCs. **G** OCR and quantitative data in M3 iPSCs sublines (M3^Low^, M3^Med^) as compared with those of N iPSCs. Data are presented as mean ± SEM, n = 3. *p < 0.05; **p < 0.01; ***p < 0.001

We have established and maintained 6 iPSCs clones from 3 different MERRF patients (M1, M2 and M3) with the m.8344A > G mutation through the collaboration (Table 1) by reprogramming skin fibroblasts of MERRF patients using retroviruses carrying OCT4, SOX2, KLF4, and GLIS1 genes. These iPSCs were characterized as previously described [12, 14]. M1 and M2 patients were from the same MERRF family [17, 22]. Only M1^Low^, M1^High^ and M2^High^ iPSCs were used in a previous study [12]. M3-iPSCs subline was generated from the skin fibroblasts of the third MERRF patient. The MERRF patients-derived iPSCs exhibited flat and tightly-packed morphology and were round shape with large nucleoli. Immunofluorescence staining showed typical expression of pluripotency markers (SEEA4 and SOX2) in all the MERRF-iPSC clones and N iPSCs (Fig. 1A). The proportions of mtDNA with the m.8344A > G mutation were ~ 50% in M1^High^, 0% in M1^Low^, ~ 60% in M2^High^, ~ 5% in M3^Low^, and ~ 30% in M3^Med1^ and M3^Med2^, respectively (Fig. 1B). M1^Low^ iPSCs, an isogenic subline of M1 iPSCs but without detectable mtDNA mutation, was used as the control of M1 iPSCs. In addition, M3^Low^, M3^Med1^ and M3^Med2^ iPSCs are another isogenic iPSC sublines with different loads of the m.8344A > G mutation. The heteroplasmy level of the m.8344A > G mutation in MERRF-iPSCs was further confirmed by next generation sequencing (NGS) analysis (Table 1). The results from the whole genome sequencing of mtDNA suggest that random segregation of the m.8344A > G mutation occurred during the reprogramming of skin fibroblasts to generate iPSCs.

**Table 1:** A list of iPSCs lines generated from patients with MERRF syndrome used in this study

iPSC lines	Original cells	mtDNA genotype	FH	m.8344A > G mutation load (%)					
				SF		iPSCs		iNSCs	
				RFLP	NGS	RFLP	NGS	RFLP	NGS
M1^Low^ iPSCs	M1 SF	m.8344A > G	Yes	97.0 ± 2.6	95.4	N.D.	N.D.	N.D.	N.D.
M1^High^ iPSCs	M1 SF	m.8344A > G	Yes	97.0 ± 2.6	95.4	47.8 ± 0.9	41.7	38.7 ± 2.5	35.9
M2^High^ iPSCs	M2 SF	m.8344A > G	Yes	47.0 ± 0.6	N.A.	57.0 ± 0.4	51.6	59.9 ± 2.4	55.4
M3^Low^ iPSCs	M3 SF	m.8344A > G	Yes	98.3 ± 1.2	85.5	5.4 ± 1.0	6.2	3.5 ± 0.6	7.3
M3^Med1^ iPSCs	M3 SF	m.8344A > G	Yes	98.3 ± 1.2	85.5	27.9 ± 0.9	25.4	27.6 ± 1.9	22.1
M3^Med2^ iPSCs	M3 SF	m.8344A > G	Yes	98.3 ± 1.2	85.5	26.5 ± 2.1	N.A.	26.0 ± 3.0	N.A.
N iPSCs	N PBMC	Wild type	No	N.A.	N.A.	N.D.	N.D.	N.D.	N.D.

*FH* family history, *N* normal, *N.A.* not available, *N.D.* not detectable, *SF* skin fibroblast

The protein expression levels of stemness genes, including TRA-1-60, SOX2 and NANOG in MERRF-iPSCs are shown in Fig. 1C. Quantitative analysis showed no consistent changes in the protein levels of stemness genes in MERRF-iPSCs harboring the m.8344A > G mutation. Our previous studies showed that m.8334A > G mutation led to a decline in the expressions of mitochondrial respiratory enzymes, especially the subunits of cytochrome *c* oxidase, and compromised mitochondrial function in the skin fibroblasts derived from patients with MERRF syndrome [22, 23]. To evaluate the effect of the m.8334A > G mutation on the bioenergetic function, the expression levels of OXPHOS proteins and mitochondrial respiratory function of MERRF-iPSCs were analyzed. As shown in Fig. 1D, MERRF-iPSCs with a high mutation load (M1^High^ and M2^High^) exhibited decreased protein level of cytochrome *c* oxidase subunit II (COX II) as compared to N iPSCs and M1^Low^. The M3 iPSC sublines with a moderate mutation load compared to the isogenic control M3^Low^ showed a compensatory increase in some of the OXPHOS proteins (Fig. 1E). A Seahorse XF^e^24 analyzer was used to measure the oxygen consumption rate (OCR) of MERRF-iPSC lines. MERRF-iPSCs, harboring a high proportion of mtDNA with the m.8344A > G mutation (M1^High^ and M2^High^), exhibited remarkable decrease of respiratory function (Fig. 1F). The basal, maximal, and ATP-coupled respiration rates of M1^High^ and M2^High^ iPSCs were significantly reduced as compared with those of the control M1^Low^. However, no significant difference in respiration rates was found between the M3 iPSC sublines, although they all had lower respiratory function as compared to the normal iPSCs (Fig. 1G).

### Impairment of mitochondrial function and altered redox status in iNSCs derived from MERRF-iPSCs

**Fig. 2:**
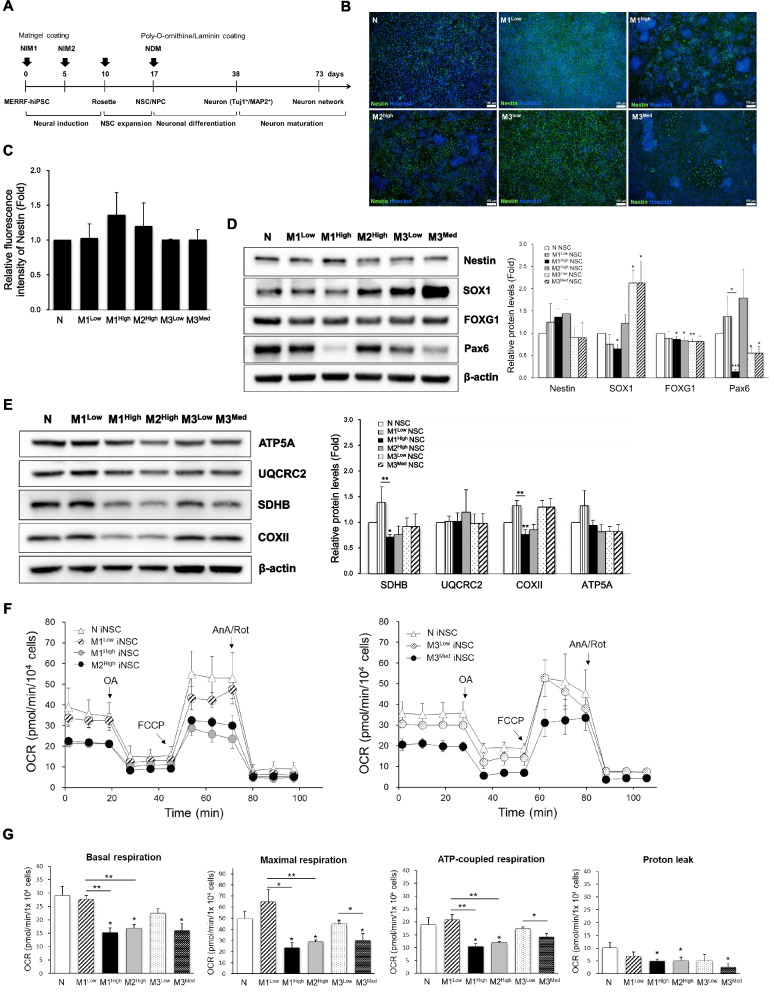
Impairment of mitochondrial respiratory function in iNSCs derived from mutant MERRF-iPSCs. **A** A schematic diagram to depict the stages for cortical neuronal differentiation of MERRF-iPSCs. **B** Characterization of neural stem cells (NSCs) derived from MERRF-iPSCs after going through the neural-lineage differentiation by the immunofluorescence staining of neural stem cell marker, Nestin (Green). Nuclei were counterstained with Hoechst 33342 (blue). Scale bars, 100 μm. **C** Quantification of the Nestin fluorescent intensity in immunofluorescence image was normalized with nuclei counts. All the data are presented as a fold change of normal iNSCs (N iNSCs). **D** The protein expression patterns of NSCs marker genes in normal and MERRF-iNSCs lines. Quantification of the proteins in Western blots was normalized with β-actin. All data displayed as a fold of N iNSCs. **E** The protein expression levels of some subunits of respiratory enzyme complexes in normal and MERRF-iNSCs. Quantification of the proteins in Western blots was normalized with β-actin. All the data displayed as a fold change of N iNSCs. **F** Mitochondrial respiration rates of normal and MERRF-iNSCs were analyzed by a Seahorse XF^e^24 extracellular flux analyzer. **G** Quantitative data of the basal respiration rate, maximal, and ATP-coupled mitochondrial respiration rates of MERRF-iNSCs (M1^Low^, M1^High^, M2^High^, M3^Low^, and M3^Med^) as compared with those of N iNSCs. Data are presented as mean ± SEM, n = 3. *p < 0.05; **p < 0.01

To establish the cell model of MERRF syndrome, MERRF-iPSCs were differentiated into mature cortical-like excitatory neurons by utilizing a 2-step neural differentiation protocol (Fig. 2A). Neural induction of normal and MERRF iPSCs was first achieved with a dual SMAD inhibition by blocking TGF-α and BMP signaling pathways to produce induced neural stem cells (iNSCs) when iPSCs were cultured in an induction medium. After 10 days, obvious neural rosettes appeared and iNSCs were harvested for morphological and phenotypic characterization. By immunofluorescence imaging MERRF-iNSCs (derived from MERRF-iPSCs) and normal iNSCs (N iNSCs) all expressed the neural progenitor cell marker Nestin (Fig. 2B and C). We further analyzed the expression of NSC-specific markers of MERRF-iNSCs by Western blots. As shown in Fig. 2D, MERRF-iNSCs exhibited typical NSC markers, including Nestin, SOX1 (SRY-Box transcription factor 1), and FOXG1 (Forkhead box protein G1). Quantitative data of Western blots and immunostaining showed no consistent changes in the protein levels of NSC markers. Although the neural-lineage characteristics were not affected by the m.8344A > G mutation in the MERRF-iNSCs, reduction in the protein level of Pax6 (Paired box protein Pax-6) was observed in M1^High^ as compared with the isogenic control M1^Low^. In addition to M1^High^, two M3 iNSCs (M3^Low^ and M3^Med^) had lower expression of Pax6 than did N iNSCs. As shown in Table 1, MERRF iPSCs-derived iNSCs had similar levels of the m.8344A > G mutation as compared with their parental iPSCs. M1^High^ iNSCs exhibited reduced levels of COXII and SDHB as compared with the M1 ^Low^ (Fig. 2E). Results of OCR assay revealed a more severe impairment of respiratory function in mutant MERRF iNSCs as compared to their isogenic controls of M1^Low^ and M3^Low^ (Fig. 2F). Quantitative analysis showed that the ATP-coupled and maximal respiration rates of M1^High^ iNSCs were reduced to 50.1% and 36.3% of that of M1^Low^ iNSCs. Moreover, M3^Med^ iNSCs showed significant decrease in the ATP-coupled respiration (81.4% of M3^Low^) and maximal respiration (66.1% of M3^Low^) (Fig. 2G).

**Fig. 3:**
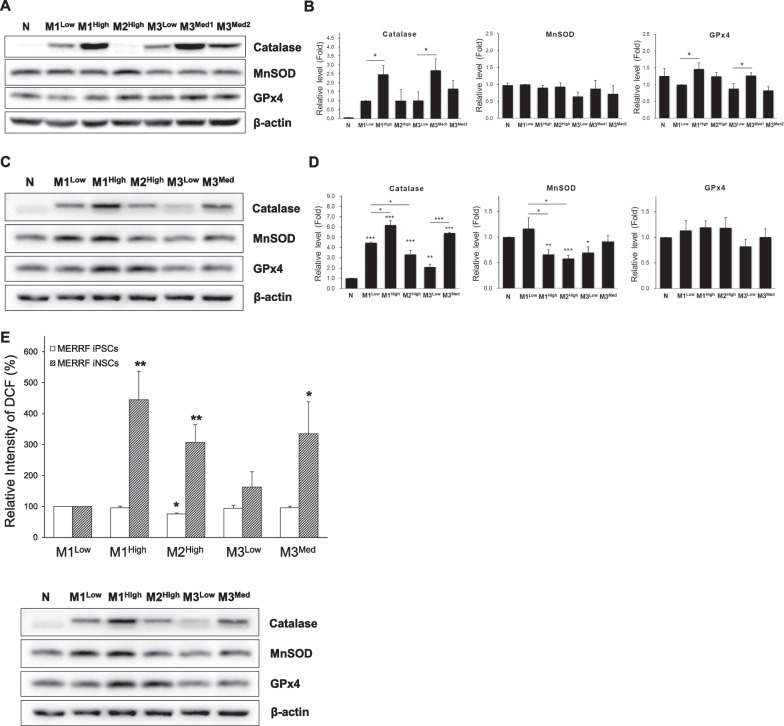
Imbalanced expression of antioxidant enzymes and accumulation of intracellular H_2_O_2_ in MERRF-iNSCs harboring the m.8344A > G mutation. **A** The protein expression levels of antioxidant enzymes in normal and MERRF-iPSCs. **B** Quantification of the proteins in Western blots was normalized with β-actin. All the data displayed as a fold of M1^Low^ iPSCs. **C** The protein expression levels of antioxidant enzymes in normal and MERRF-iNSCs. **D** Quantification of the antioxidant enzyme proteins in iNSCs was normalized with β-actin. All the data are expressed as the fold change of those of N iNSCs. **E** Intracellular levels of H_2_O_2_ in MERRF-iPSCs and MERRF-iNSCs were analyzed by staining cells with DCFH-dA and were quantified in comparison with those of the control (M1^Low^ iPSCs and M1^Low^ iNSCs), respectively. The fluorescence intensity is expressed as the percentage change of M1^Low^. Data are presented as mean ± SEM, n = 3. *p < 0.05; **p < 0.01; ***p < 0.001

To further examine the effects of m.8344A > G mutation on cellular redox status, the intracellular levels of ROS and antioxidant enzymes in MERRF-iPSCs and iNSCs were determined, respectively. Western blot analysis revealed elevated expression of catalase but no concurrent change of MnSOD, in both iPSCs (Fig. 3A and B) and iNSCs (Fig. 3C and D) of M1^High^ and M3^Med^ as compared with their isogenic control. A reduction in the protein level of MnSOD was observed in M1^High^ iNSCs (Fig. 3D). These results indicate imbalanced expression of antioxidant enzymes in mutant MERRF iPSCs and iNSCs. The intracellular levels of H_2_O_2_ in MERRF-iPSCs and MERRF-iNSCs were then analyzed by the DCFH-dA staining of cells. Figure 3E shows the relative H_2_O_2_ levels of MERRF-iPSCs and MERRF-iNSCs as compared with their respective control (M1^Low^). The fluorescence intensity of DCF in these two groups were respectively quantified in comparison with those of the control (M1^Low^ iPSCs and M1^Low^ iNSCs) and are expressed as the percentage change of M1^Low^. The results indicate a concurrent increase in the intracellular H_2_O_2_ levels in mutant MERRF iNSCs as compared to the control M1^Low^ (445.5% for M1^High^, 307.4% for M2^High^ and 335.5% for M3^Med^), but not in the parental iPSCs. This implies a cell-specific effect of the m.8344A > G mutation on the accumulation of H_2_O_2_ in iNSCs. These results suggest that MERRF iPSC-derived iNSCs recapitulated phenotypic impairment of mitochondrial function, ROS overproduction, and dysregulation of the antioxidant defense system.

### Generation and characterization of cortical-like neurons derived from neural-lineage differentiation of MERRF-iPSCs

**Fig. 4:**
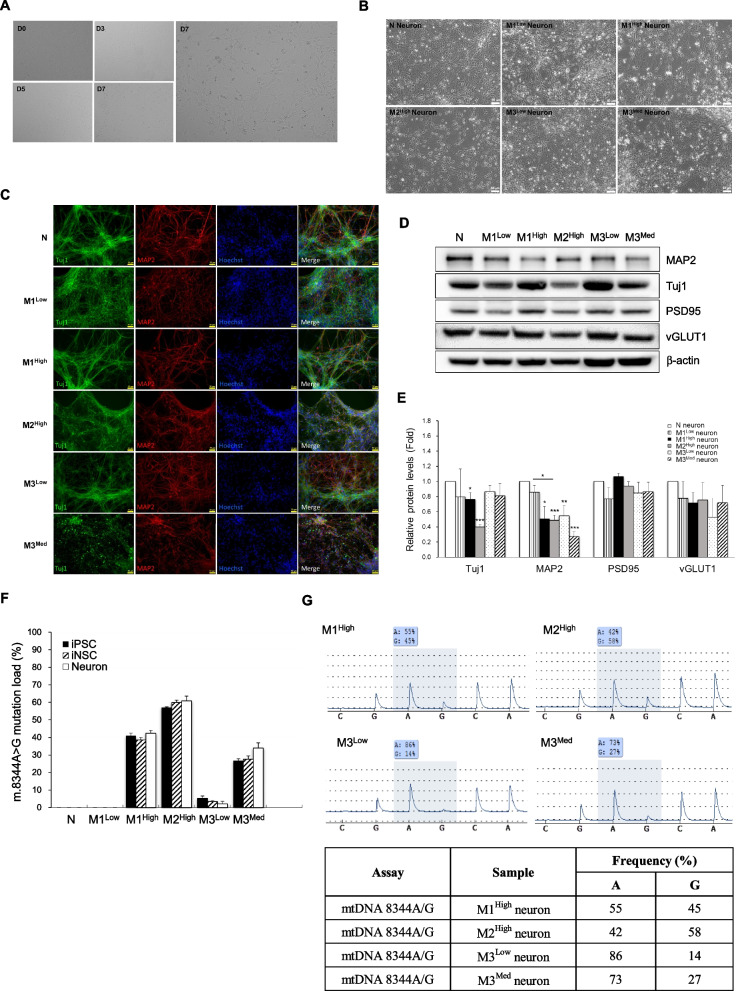
Generation and characterization of cortical-like neurons derived from MERRF-iPSCs undergoing neural-lineage differentiation. **A** Morphological change in the normal iNSCs at early stage of neuronal differentiation was observed at the indicated time points. **B** Morphological characterization of the iNSCs-derived neurons at day 14 of neuronal differentiation. Scale bars, 50 μm. **C** Phenotypic characterization of MERRF iNSCs-derived cortical-like neurons after 3 weeks of differentiation by the immunofluorescence staining with pan neuron markers, MAP2 (red), Tuj1 (green), and Hoechst 33342 (blue). Scale bars, 50 μm. **D** The protein expression levels of neuron-specific markers in normal and MERRF neurons after 3 weeks of differentiation. **E** Quantification of the proteins in Western blots was normalized with β-actin. All the data displayed as a fold change of normal neurons (N neurons). **F** Preservation of the heteroplasmy levels of the m.8344A > G mutation in MERRF neurons and the parental MERRF-iNSCs and MERRF-iPSCs. The proportion of mtDNA with the m.8344A > G mutation was quantified by PCR–RFLP. **G** The mutation level of m.8344A > G in MERRF neurons was confirmed by pyrosequencing. Data are presented as mean ± SEM, n = 3. *p < 0.05; **p < 0.01; ***p < 0.001

Development of human cortical neurogenesis from iPSCs is essential for the establishment of an in vitro cell model of cortical neuron-related disorders [24]. In order to clarify the pathology and underlying mechanism of neurological disorder in mitochondrial disease, MERRF iNSCs were differentiated toward cortical-like neurons. Normal and MERRF-iNSCs were cultured in a defined medium to undergo terminal neuronal differentiation. Schematic diagram shown in Fig. 2A illustrated the time course of the in vitro differentiation and terminal maturation of cortical glutamatergic neurons derived from MERRF-iNSCs. The maturation of glutamatergic neurons was determined at different stages, by evaluating neurite outgrowth, synapse formation and maturation, and neuronal network connectivity. After 7–21 days of differentiation, neurons were harvested for characterization of mature neurons by immunofluorescence staining and Western blot analysis of marker proteins. At the early stage of the neural differentiation, neurite outgrowth was observed to form axons and dendrites. As shown in Fig. 4A, the neurites were observed in N iNSCs after 7 days of neuronal differentiation. Figure 4B shows the morphological alterations in normal and MERRF-iNSCs after 14 days of differentiation. The results revealed that normal neuron (N neuron) and M3^Low^ neuron exhibited higher neurite density than did mutant MERRF neurons (M1^High^, M2^High^ and M3^Med^ neurons). The phenotypic characterization of glutamatergic neurons derived from iNSCs was performed by immunofluorescence staining of neuron-specific markers, β-tubulin III (Tuj1) and microtubule-associated protein 2 (MAP2), which can be used to trace neurite outgrowth in the axon and dendrite, respectively. The immunofluorescence staining images showed that most of the normal and MERRF neurons were Tuj1^+^ and MAP2^+^ cells (Fig. 4C) and exhibited typical neural morphology. However, mutant MERRF neurons, especially M3^Med^, had increased cell death at early stage of neuronal differentiation. At the middle stage of differentiation, synapses were formed and progressively matured to establish functional excitatory connections through synaptic transmission. We next assessed the protein expression profile of the neuron-specific genes in MERRF patient’s iPSCs-derived neurons after 21 days of glutamatergic neuronal differentiation. As shown in Fig. 4D, all neurons expressed pan neuron markers including Tuj1, MAP2, postsynaptic density protein 95 (PSD95), and vesicular glutamate transporter 1 (vGLUT1), a glutamatergic neuron-specific marker. The quantitative results showed that M1^High^ and M2^High^ neurons exhibited lower Tuj1 expression levels and all mutant neurons had reduced levels of MAP2 as compared with normal neurons (Fig. 4E). The linear regression analysis showed a negative correlation between the Tuj1 level and the mutation load of m.8344A > G (Additional file 1: Table S1). We next determined the heteroplasmy levels of the m.8344A > G mutation in MERRF neurons by PCR–RFLP (Fig. 4F), and further confirmed by pyrosequencing (Fig. 4G). The results indicate that mutant MERRF iNSCs-derived neurons possessed similar proportions of the m.8344A > G mutation as compared with the original iPSCs and iNSCs, whereas M1^Low^ had undetectable mtDNA mutation. Collectively, these findings suggest that both normal and MERRF-iNSCs can be differentiated into cortical-like excitatory neurons, which implied that the m.8344A > G mutation did not affect the initial cortical neuronal differentiation but caused a decrease in the expression levels of neuron-specific cytoskeleton proteins such as Tuj1 and MAP2.

### Attenuated spontaneous electrical activity in MERRF iPSCs-derived neurons with the m.8344A > G mutation

**Fig. 5:**
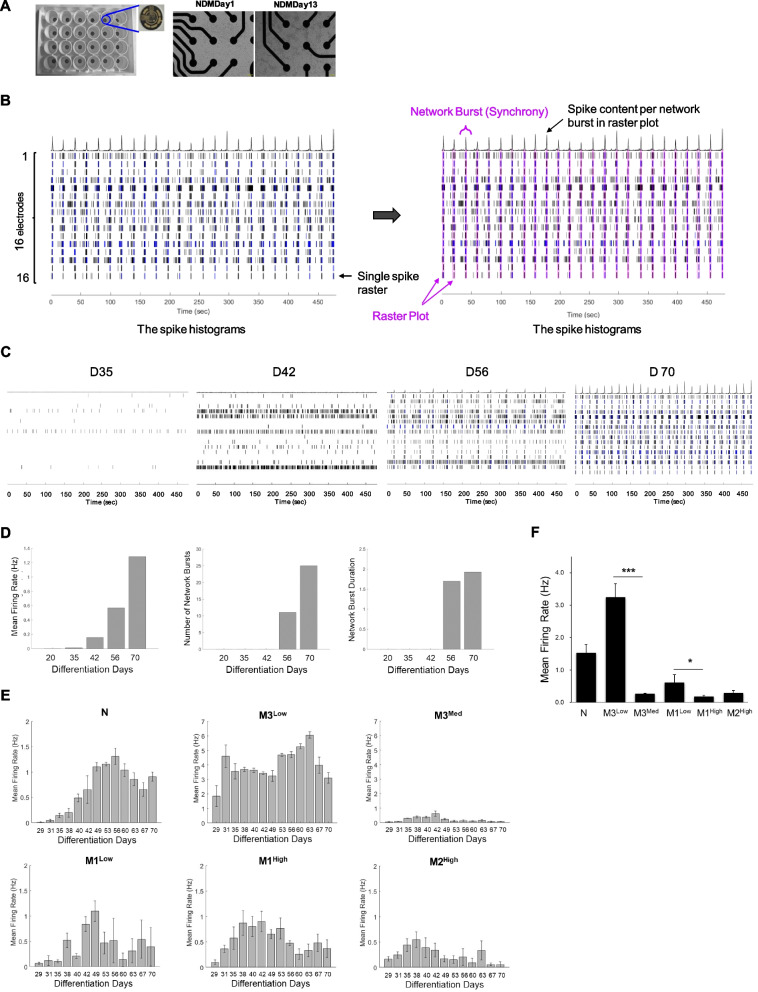
Electrophysiological assessment of spontaneous activity in iPSCs-derived cortical-like neurons. **A** The image of a 24-well Axion microelectrode array (MEA) containing 16 electrodes and used for measuring the electrophysiological activity of neurons. iNSCs were cultured on MEA, and spontaneous excitatory activity was recorded during neuronal differentiation. **B** A representative raster trace of M3^Low^ neurons in a well measured from an 8-min recording at day 70 of differentiation. Each horizontal row represents an electrode in the well. The spike histogram shows a synchronous network burst recording plotted over all electrodes (purple bar). **C** The alteration of spontaneous neuron activity in M3^Low^ neurons at indicated time of differentiation (day 35, day 42, day 56, and day 70). **D** A plot represents mean firing rate (MFR), network burst numbers, and burst duration measured by the MEA. **E** Normal and MERRF-iNSCs were replaced in the MEA and allowed to undergo neuronal differentiation for 10 weeks, the spontaneous electrical activity of neurons was recorded over a period of 8 min. The plot represents the mean firing rate in neurons measured from day 29 to day 70 after neuron differentiation. Data were calculated as the average of 3 to 4 replicate wells of each neuron group in the experiment. **F** Mean firing rate in N and MERRF neurons at 7-week maturation. Data are presented as mean ± SEM, n = 3. *p < 0.05; **p < 0.01; ***p < 0.001

At the late stage, neuronal network between mature cortical neurons is fully functional to support spontaneous and synchronized communication. To evaluate the functional maturation of excitatory glutamatergic neurons derived from iNSCs, electrophysiological properties of normal and MERRF neurons were examined during neuronal maturation. The electrophysiological recordings were performed using the microelectrode arrays (MEAs) system (Axion Biosystems, Atlanta, GA, USA) [21]. The normal and MERRF-iNSCs were plated on a MEA chip (Fig. 5A) and were allowed to undergo neuronal differentiation for more than 70 days, and the spontaneous action potential on the 16 electrodes of each well was monitored over time and visualized by a spike raster. An array-wide raster trace shown in Fig. 5B represents the spontaneous firing patterns of single well neurons 70 days after differentiation. The electrode signal provides the individual neuron firing and bursting, and connective signal between multiple electrodes can provide network bursting and synchrony. We collected electrophysiological activity data for 8 min from neuronal networks by MEA every two days starting from day 20 after neuronal differentiation (3 to 4 replicates per experiment). The spike histograms shown in Fig. 5C revealed that the spontaneous activity of glutamatergic neurons was gradually increased with the differentiation, as indicated by the average spike numbers measured over 8 min. The representative data of spontaneous firing patterns at 35, 42, 56, 70 days after differentiation showed a steady increase in the neuronal activity towards maturation (Fig. 5C). The parameters showed that the mean firing rate, the numbers of network burst and burst duration were significantly increased in a time-dependent manner (Fig. 5D). Moreover, the functional network behaviors in neurons occurred at 7–8 weeks of neuronal maturation. We further assessed the effect of mitochondrial dysfunction on the excitatory neuronal activity by measuring the spontaneous electrical activity with the MEA system in normal and MERRF neurons over 10 weeks of differentiation. Figure 5E shows the time-dependent changes in mean spike firing rate in both MERRF and normal neurons from 29 to 70 days after neuronal differentiation. The number of activity-response electrodes (> 5 spikes/min) was increased with maturation, reaching almost 100% on 4 weeks in both MERRF and normal neurons. However, an immature neuronal network was observed in MERRF neurons harboring the m.8344A > G mutation. The quantitative data shown in Fig. 5F indicated that at 7 weeks after maturation, M1^High^, M2^High^ and M3^Med^ had significantly lower mean firing rate as compared with the controls M1^Low^ and M3^Low^, respectively.

### Impairment of synaptic plasticity in mature MERRF neurons harboring the m.8344A > G mutation

**Fig. 6:**
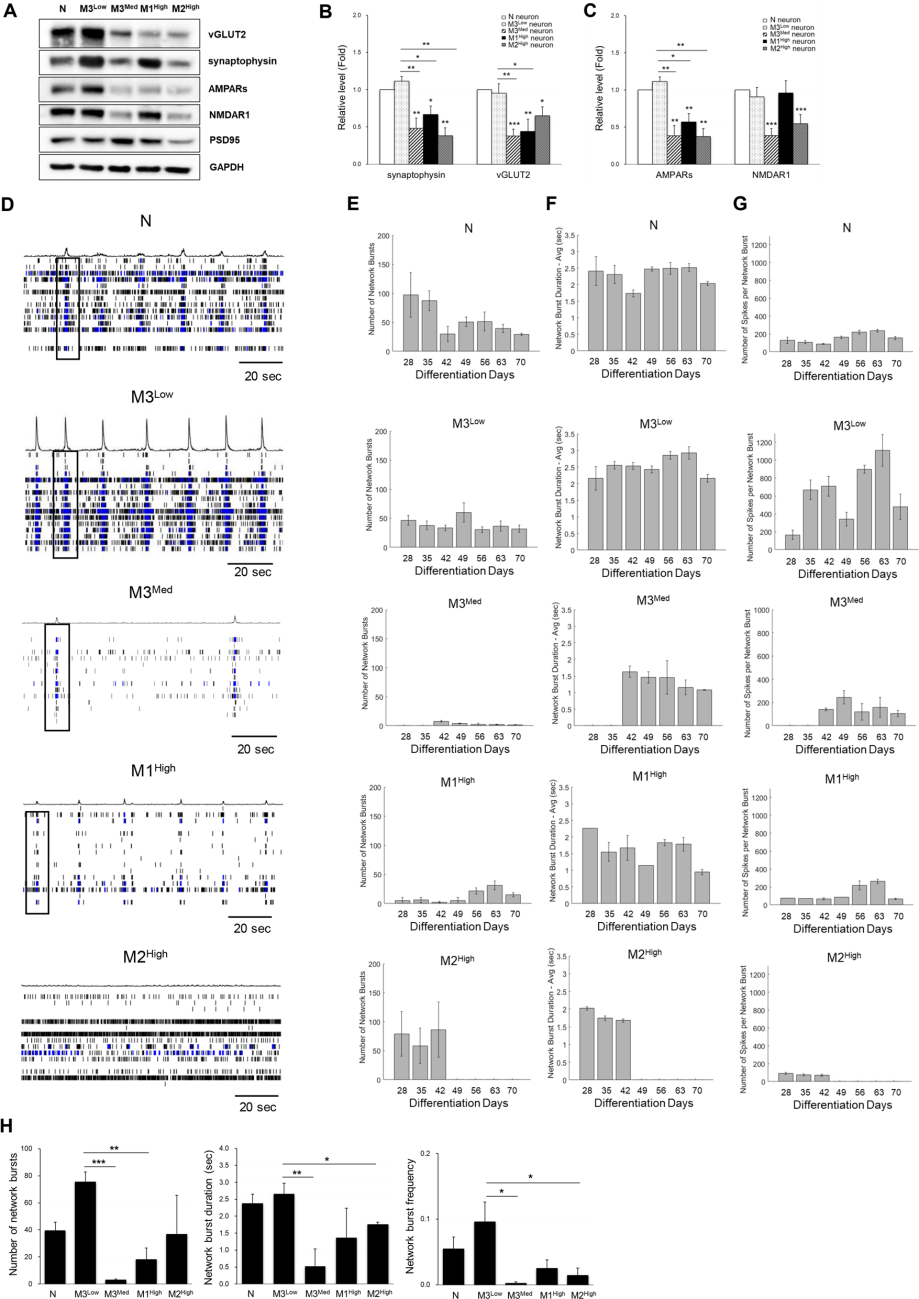
Synaptic defects in MERRF neurons harboring the m.8344A > G mutation. **A** The protein expression levels of presynaptic vesicular proteins and excitatory glutamate receptors in normal and MERRF neurons. **B** Quantitative data of the synaptic vesicular proteins, synaptophysin and vGLUT2. **C** Quantitative data of the excitatory receptors, AMPARs and NMDARA. Quantification of the proteins in Western blot was normalized with β-actin. All data are presented as the fold change of that in N neurons. **D** Representative image of spike histograms showing the spontaneous network bursting in N neurons and MERRF neurons from 2-min recording plotted over all electrodes. Temporal changes in **E** the number of network bursts, **F** network burst duration, and **G** the number of spikes per network burst during 10 weeks of differentiation. Data are presented as the average of 3–4 replicate wells of each neuron group in the experiment. **H** Quantitative parameters in N neurons and MERRF neurons at 7 weeks of maturation. Data are presented as mean ± SEM, n = 3. *p < 0.05; **p < 0.01; ***p < 0.001

Formation of synaptic vesicles and functional synapse sustain neurotransmitter release is important for neuronal activity [25]. Synaptophysin plays a crucial role in the formation, maintenance, and plasticity of synapses. It is primarily located in the presynaptic terminals of neurons and regulates the fusion of the synaptic vesicles with the presynaptic membrane modulating neurotransmitter release. To investigate the effect of the m.8344A > G mutation on the synaptic function and its involvement in the neuronal dysfunction, we next examined the expression levels of synaptic vesicular proteins and excitatory glutamate receptors of dendrite synapses in normal and 4 MERRF neuron cell lines. We observed a reduction in the protein levels of synaptophysin and vesicular glutamate transporter 2 (vGLUT2) in mutant MERRF neurons (Fig. 6A). Quantitative analysis showed that both synaptophysin and vGLUT2, were remarkably decreased in M3^Med^, M1^High^, and M2^High^ as compared to either normal or M3^Low^ neurons (Fig. 6B). The protein level of synaptophysin in MERRF neurons was negatively correlated with the level of m.8344A > G mutation (Additional file 1: Table S1). Additional immunostaining experiments revealed that the synaptic vesicle density within the presynaptic axon was decreased in MERRF neurons harboring the m.8344A > G mutation (Additional file 2: Figure S1). This observation implies that the loss of synaptic proteins might affect the synapse structure and neurotransmitter transport. We also determined the protein levels of excitatory neuron receptors in these iPSC-derived neurons and found that AMPARs (alpha-amino-3-hydroxy-5-methyl-4-isoxazolepropionic acid receptors), the ionotropic glutamate receptors, were significantly reduced in the 3 mutant MERRF neurons (M3^Med^, M1^High^, and M2^High^) as compared with the normal and M3^Low^ neurons (Fig. 6A and C). Together, the above findings suggest that a loss of synaptic proteins is involved in the dysfunction of presynaptic vesicles and postsynaptic excitatory receptors, which may lead to synaptic deficiency in MERRF neurons harboring the m.8344A > G mutation.

Synaptic dysfunction has been shown to affect the connectivity and plasticity of neuronal networks [26–28]. To explore whether synaptic defect is associated with neuron network activity in mutant MERRF neurons, we examined network bursts in MERRF and normal neurons. Network bursts are coordinated firings of a population of neurons characterized by brief bursts of high-frequency activity. This burst can enhance the synchrony of neuronal firing and promote the formation of new connections between neurons. The spike histograms in Fig. 6D revealed that after 7 weeks of differentiation, both normal and control (M3^Low^) neurons exhibited synchronous network bursts, a critical characteristic of mature neuronal network, whereas the attenuation of network bursts was observed in MERRF neurons with higher levels of the m.8344A > G mutation (M3^Med^, M1^High^, and M2^High^ neurons). Figure 6E–G show the time-dependent changes in the number of neuron network bursts, network burst duration, and the number of spikes per network burst, respectively, during 4–10 weeks of differentiation. During differentiation, the spontaneous network bursting reproducibly observed in normal and M3^Low^ neurons, however, was delayed and declined dramatically in M3^Med^, M1^High^, and M2^High^ neurons (Fig. 6E). The representative parameters in Fig. 6E–G showed an asynchronized burst activity in M2^High^ neurons after the 7 weeks of differentiation. The quantitative data revealed that the number of network bursts, burst duration, and the frequency of network burst were significantly decreased in MERRF neurons harboring higher levels of the m.8344A > G mutation as compared with those of the normal and M3^Low^ neurons at 7 weeks of differentiation (Fig. 6H), which indicate phenotypic impairment of spontaneous and synchronous neuronal activities in mutant MERRF neurons.

**Fig. 7:**
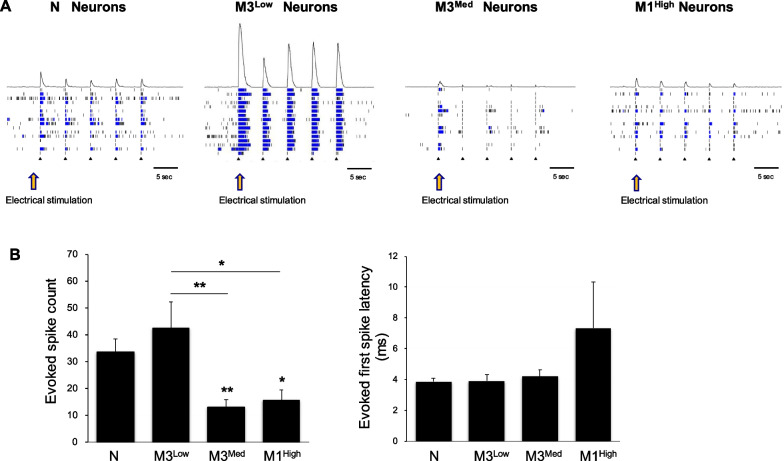
Attenuation of evoked potential-stimulated excitability in MERRF neurons harboring the m.8344A > G mutation. **A** The spike histograms shows the evoked electrical activity of neurons during 5 times of continuous current stimulation in neurons after 10 weeks of differentiation. **B** A plot represents the number of evoked potential-stimulated spike and evoked first spike latency in MERRF neurons compared to those of N and M3^Low^ neurons. Data are presented as mean ± SEM, n = 3. *p < 0.05; **p < 0.01

To assess the functions of ionotropic glutamate receptor and synaptic plasticity in mature neurons, three lines of MERRF neurons (M3^Low^, M3^Med^, M1^High^) and normal neurons were stimulated with an electric current to examine the alteration of network excitability and connectivity. The spike histograms showed the evoked potential-stimulated activity of neurons during 5 times of continuous current stimulation in neurons (Fig. 7A). We found that the electric stimulation promoted the excitability (increased spike numbers and network duration) and synchronous network activity in normal and M3^Low^ neurons, but only weak response was observed in M3^Med^ and M1^High^ neurons. As shown in Fig. 7B, the evoked spike number (evoked spike count) was decreased in mutant MERRF neurons as compared with those of the normal and M3^Low^ neurons, but no significant difference in the evoked first spike latency, which means the time it takes for a neuron to fire its first action potential in response to a stimulus. These findings suggest that iPSCs-derived cortical glutamatergic neurons exhibited typical neuronal phenotype, functional synaptic activity and synchronous neuron network. However, the synaptic response to electric stimulation was decreased in the MERRF neurons harboring higher levels of m.8344A > G mutation. These results together suggest that the pathogenic m.8344A > G mutation can cause synaptic impairment, which in turn impede neuron network maturation and synaptic plasticity in the excitatory neurons derived from iPSCs of MERRF patients.

## Discussion

MERRF patient’s iPSCs-derived iNSCs that recapitulated the phenotype of impaired mitochondrial bioenergetic function and accumulation of H_2_O_2_ were successfully established for the first time as a neuronal model of MERRF syndrome (summarized in Table 2 and Additional file 1: Table S1). The electrophysiological properties of neurons have been widely studied to understand the pathophysiology of brain injury and neurodegenerative diseases [29, 30]. We used the MEA electrophysiological platform to investigate the neuronal behaviors of MERRF iPSCs-derived cortical-like neurons. Our findings revealed the pathological effect of the m.8344A > G mutation on neuronal activity and synaptic plasticity attributed by a decrease in the expression of presynaptic proteins and attenuated excitatory receptor in the MERRF neurons (Fig. 6B and C).

**Table 2:** The effects of m.8344A > G mutation on the biological function of neural cells derived from MERRF-iPSCs

	M1 MERRF patient			M3 MERRF patient		
	M1^High^			M3^Med^		
	iPSC	iNSC	Neuron	iPSC	iNSC	Neuron
m.8344A > G mutation level (%)	41.1 ± 1.4	38.7 ± 1.4	42.5 ± 1.7	28.8 ± 1.1	27.6 ± 1.9	34.0 ± 2.9
OXPHOS^a^						
COXII	↓	↓		↑	ND	
SDHB	ND	↓		↑	ND	
ATP5A	ND	ND		ND	ND	
Respiration rate^a^						
Basal	↓	↓		ND	ND	
ATP-coupled	↓	↓		ND	↓	
Maximal	↓	↓		ND	↓	
Proton leak	ND	ND		ND	ND	
ROS levels^a^	ND	↑	↑	ND	↑	↑
Antioxidant enzymes^a^						
Catalase	↑	↑		↑	↑	
MnSOD	ND	↓		ND	ND	
GPx4	↑	ND		↑	ND	
Characteristic markers^a^						
Stemness markers	ND			ND		
NSC specific markers		ND			ND	
Pan neuron markers			ND			ND
Neuron cell death^a^			↑			↑
Synaptic vesicular proteins^b^						
Synaptophysin			↓			↓
vGLUT2			↓			↓
Excitatory receptors^b^						
AMPARs			↓			↓
NMDAR1			ND			↓
Neuronal function						
Spontaneous MFR^a^			↓			↓
Neuron network^b^			↓			↓
Evoked response^b^			↓			↓

a: compared with isogenic control cells of subline; b: compared with M3^Low^ neuron

*AMPARs* α amino-3-hydroxy-5-methyl-4-isoxazolepropionic acid receptors, *ATP5A* ATP synthase α subunit, *SDHB* succinate dehydrogenase complex iron sulfur subunit b, *COXII* cytochrome *c* oxidase subunit II, *GPx4* glutathione peroxidase 4, *iNSC* induced neural stem cells differentiated from iPSCs, *MFR* mean firing rate, *MnSOD* manganese-dependent superoxide dismutase, *ND* no difference, *NMDAR1* N-methyl-d-aspartate receptor 1, *vGLUT2* vesicular glutamate transporter 2. ↓, decreased; ↑, increased

The MEA system is a powerful tool used in neuroscience research to investigate the activity of neuronal networks in vitro [31]. This system can record the electrical activity of multiple neurons simultaneously and in real-time on the surface of the array, which provides researchers a technique for neurotoxicity screening and investigating the underlying mechanisms of a wide range of neurological disorders [9, 32] including epilepsy, Alzheimer’s disease, Parkinson’s and mitochondrial diseases. Experimental evidence [31] indicates that some parameter of MEA analysis can be directly compared to the electroencephalography (EEG) data obtained from the human brain. EEG is a non-invasive and widely available diagnostic tool to measure the electrical activity in the brain, which reflects the brain dysfunction commonly seen in neurological disorders and brain injury. The EEG signature of MERRF syndrome showed a characteristic pattern of neuronal abnormalities, which include generalized reduction of neuronal activity and epileptiform discharges due to abnormal electrical activity in the brain and predisposition to seizures. According to the medical records, M1 patient exhibited diffuse cortical dysfunction and focal spiking temporal areas with multiple decreases in amplitude and delayed latencies. Focal or generalized decrease in brain activity indicates a disruption in neuronal communication. The electrophysiological data obtained in this study truly reflect these neuropathological defects in M1^High^ neurons (Figs. 5, 6, 7). Therefore, the application of MEA to measure neuronal networks in MERRF iPSC-derived neurons, combined with pharmacological manipulations and their comparison with EEG data, has great potential to improve our understanding of neuropathogenesis of the mitochondrial disease and provide new information for the development of new therapies.

In the early stages of neurogenesis, we observed that mutant MERRF neurons (M1^High^ and M2^High^ and M3^Med^) exhibited slower neurite outgrowth and lower neurite density than did N and M3^Low^ neurons (Fig. 4B). Although MERRF patient’s iPSCs-derived neurons exhibited typical neural phenotype, M2^High^ neurons with a higher mutation load (60.8 ± 2.7%) showed decreased expression levels of Tuj1 as compared with normal neurons (Fig. 4D and E). Despite the quantitative analysis revealed a negative correlation between the Tuj1 expression level and mtDNA mutation load in MERRF neurons (Additional file 1: Table S1), Tuj1 expression in M2^High^ neurons showed a noticeable decline than did M1^High^ neurons (42.5 ± 1.7% of mutation). The diversity of nuclear genetic background among different patients may affect the level of Tuj1 besides the m.8344A > G mutation. It is well known that Tuj1 is essential for axonal and dendritic growth, its expression has been reported to be sensitive to neuronal activity change [33]. Therefore, the correlation between the expression levels of these proteins and neurite outgrowth/synaptic function in MERRF neurons warrants further investigation in the future.

Dysfunction of mitochondria has been shown to affect dendritic morphology and synapse function [34]. Synaptic vesicles are responsible for the transport of neurotransmitters to the synaptic cleft and thereby propagate signals between neurons. Synaptophysin is a glycoprotein and involved in the trafficking and recycling of the synaptic vesicles to regulate neurotransmitter release. The vesicular glutamate transporters (vGLUTs) are a family of membrane transporter that achieves excitatory glutamate transport from the cytoplasm into synaptic vesicles for store. The stored glutamate is then released from the vesicles into the synaptic cleft, where it binds glutamate receptors (e.g., AMPAR and NMDAR) on the postsynaptic membrane, leading to depolarization of the postsynaptic neuron and the initiation of an action potential. This process is critical for normal synaptic transmission and communication between neurons. The reduction in the presynaptic vesicular membrane proteins synaptophysin and vGLUT2 in mutant MERRF neurons M1^High^, M2^High^, and M3^Med^ (Fig. 6A and B) may attenuate the glutamate transport and release in neurons. The reduction of synaptophysin level in patients with Alzheimer’s disease [35] and Parkinson’s disease [36] has been reported to be attributed by the synapse loss and neuronal cell death. The synaptophysin decrease in the prefrontal cortex was demonstrated to be associated with a disruption in the balance between excitatory and inhibitory neurotransmission in schizophrenia [37]. The findings suggest that synaptophysin levels can serve as a marker of synaptic density to assess synaptic plasticity in various neurological disorders. Dysfunction of vGLUT2 has also been implicated in a variety of neurological and psychiatric disorders [38]. We found that the MERRF neurons with higher levels of the m.8344A > G mutation exhibited attenuated spontaneous electrophysiological function and network plasticity (Figs. 5F, 6H, and 7B), which truly reflect synaptic defects caused by the decrease of synaptophysin and vGLUT2.

Synaptic plasticity is the ability of synapses to change their strength and connectivity in response to neuronal activity, and is crucial for learning and memory. The mechanisms underlying synaptic dysfunction are complex and multifactorial, and the ubiquitin–proteasome degradation of proteins may contribute to the downregulation of synaptophysin [39–41]. Mitochondrial dysfunction and oxidative stress have been shown to have a significant impact on synaptic plasticity by reducing the ability of neurons to generate and sustain synaptic activity, altering the balance between excitatory and inhibitory neuronal signalling pathways, and increasing the susceptibility of neurons to excitotoxicity [42, 43]. ROS are important physiological mediators of neuronal plasticity, but excess accumulation of ROS in the brain can be detrimental to neuron activity by impairing synaptic plasticity [44]. It was reported that oxidative stress is involved in synaptic loss and downregulation of synaptophysin in Alzheimer’s disease [45]. The mitochondrial stress-induced synaptic damage was associated with the oxidative stress-mediated activation of p38 MAPK, and treatment with a p38 MAPK inhibitor improved neurogenesis and increased the expression levels of synaptophysin and PSD-95 in cybrids derived from a patient with Alzheimer’s disease [45]. Oxidative stress can also lead to the activation of inflammatory pathways, which can further exacerbate synaptic dysfunction [46]. In this study, we observed a significant increase in the intracellular levels of H_2_O_2_ in iPSCs-derived iNSCs (Fig. 3E) and neurons harboring the m.8344A > G mutation (Additional file 3: Figure S2). The overproduction of ROS by dysfunctional mitochondria in MERRF patient iPSCs-derived neurons may in turn cause the loss of neuronal plasticity or synaptic activity.

Several lines of evidence indicate the activation of Ca^2+^/calmodulin-dependent protein kinase II (CaMKII) is crucial for the regulation of synaptic plasticity in hippocampal neurons [47, 48]. CaMKII regulates the stability of AMPA receptors (AMPARs) in postsynaptic cell membrane. The interaction between AMPARs and CaMKII plays a critical role in the induction and maintenance of long-term potential (LTP) [49, 50]. AMPARs are highly concentrated in the excitatory synapses at dendrites, which mediate the rapid excitatory neurotransmission contributing to the changes in synaptic strength [51]. The recruitment and half-life of AMPARs at synapses are tightly regulated by a complex interplay of various mechanisms, including endocytosis [51, 52], synaptic activity, and long-term depression (LTD) [53], which is increasingly recognized as a crucial aspect of excitatory synapse maturation and synaptic strength regulation. Endocytosis is a key process that regulates the number of AMPARs at the synapse. When the postsynaptic membrane is stimulated by glutamate released from the presynaptic terminal, AMPARs are rapidly recruited to the synapse from the extra-synaptic pool of receptors. However, prolonged or excessive synaptic activity can trigger the internalization of AMPARs via endocytosis [52], which results in a decrease in the number of receptors at the synapse. Furthermore, low-frequency stimulation (LFS) can trigger an LTD which corresponds to a long-term reduction in synaptic transmission, resulting in the weakening of synapses and a decrease in the number of AMPARs at the synapse [53]. This decrease in AMPARs is thought to occur via the dephosphorylation of AMPAR subunits, which promotes the internalization and degradation of AMPARs through endocytosis. In the present study, we found that aberrant synaptic network activity was accompanied by the depletion of AMPAR proteins in the MERRF iPSCs-derived neurons (Fig. 6A and C), which may be associated with the dysregulation of recruitment and stability of postsynaptic AMPARs.

Clonal variation in iPSC experiments is an important issue as it can lead to variability in iPSCs differentiation potential and cell fate outcome, which may affect experimental reproducibility. Differences between donors, genetic stability and experimental variability contribute to iPSCs variation. Genetic instability has been considered to affect the quality of human iPSCs by causing functional deficiencies, which have limited the therapeutic use of autologous iPSCs. Studies have shown that random segregation of mutant mtDNA occurs during iPSCs reprogramming and mtDNA genotype instability during clonal expansion of iPSCs [12, 54]. The heteroplasmy level of m.8344G A > G mutation of each of the iPSCs and iNSCs as well as the derived neurons were determined before and after use for experiments conducted in this study. Given that the instability of mutant mtDNA affects the quality of patient iPSCs, analysis of mtDNA mutation and new variants in iPSCs subculture should be incorporated in the validation to obtain fully certified iPSCs for mitochondrial disease modeling. Understanding and accounting of clonal variation in iPSCs experiments is also important for accurate interpretation of experimental results and ensuring the reproducibility of findings before applications of iPSCs in disease modeling and drug screening.

## Conclusion

In conclusion, we have established patient-specific excitatory neurons from MERRF-iPSCs that retained the m.8344A > G mutation and exhibited phenotypic impairment of neuronal properties. Neuropathological investigations expanded our understanding of the neurological defects in patients with MERRF syndrome, which is manifested at least partly by the presynaptic protein loss of vesicular proteins and attenuation of postsynaptic glutamate receptors density. Electrophysiological signatures reflect abnormal synchronous activity and irregular synaptic connectivity in cortical networks of MERRF iPSCs-derived neurons, which are consistent with the clinical features often observed in MERRF patients. Future studies are warranted to verify the role of the m.8344A > G mutation-elicited oxidative stress in the synaptic protein loss and dysregulation of neuronal plasticity in the neurons derived from MERRF patients. Current treatment of mitochondrial diseases is limited to symptomatic improvement, which cannot remedy the biochemical defects caused by mtDNA mutations. The combined use of MERRF iPSCs-derived neurons and the MEA electrophysiological recording platform provide us a new approach for pathophysiological study of MERRF syndrome and for screening of new drugs to treat the mitochondrial disease.

### Supplementary Information

**Additional file 1: Table S1.** The relationship between the heteroplasmy level of m.8344A > G mutation and its effects on the biological functions of neural cells derived from MERRF-iPSCs.

**Additional file 2: Figure S1.** Decreased density of synaptic vesicle in the axon of neurons derived from MERRF-iPSCs harboring the m.8344A > G mutation. Distribution of synaptic vesicle in neurons 3 weeks after differentiation was analyzed by the immunofluorescence staining with antibodies against synaptophysin (red), Tuj1 (green), and Hoechst 33342 (blue). Scale bars, 20 μm.

**Additional file 3: Figure S2.** Correlation of intracellular H_2_O_2_ level and mtDNA mutation heteroplasmy in cortical neuron derived from MERRF-iPSCs harboring the m.8344A > G mutation. (A) Intracellular levels of H_2_O_2_ in normal and MERRF neurons on day 21 of neuronal differentiation were analyzed by the DCFH-dA staining of cells. The fluorescence intensity of DCF was quantified compared with that of M3^Low^ control and displayed as the percentage change of M3^Low^ neurons. Data are presented as mean ± SEM, n = 3. *: p < 0.05; **: p < 0.01; ***: p < 0.001. Cells were all subjected to three independent experiments except M2^High^, which was used to perform only two independent experiments. (B) Linear regression analysis of the m.8344A > G mutation load and the H_2_O_2_ levels in MERRF neurons. Regression of a continuous quantitative variable of mutation load with the average DCF fluorescence intensity in neurons was performed using an Excel linear regression model. (R^2^ = 0.844, p = 0.027, regression coefficient = 2.191).

## Data Availability

The experimental data, tables, and figures presented in this manuscript have not been published and are not considered for publication elsewhere. The authors have read the manuscript and agreed to submit it to JBS for publication in this special issue after peer review. The datasets used and/or analyzed during the current study are available from the corresponding author on request.

## Abbreviations

AA: Antimycin A
AMPARs: Alpha-amino-3-hydroxy-5-methyl-4-isoxazolepropionic acid receptors
ATP5A: ATP synthase α subunit
CaMKII: Calcium/calmodulin-dependent protein kinase II
COX II: Cytochrome *c* oxidase subunit II
CRE: CAMP response element
CREB: CAMP response element binding protein
DCFH-DA: 2,7-Dichlorofluorescin diacetate
EEG: Electroencephalography
FOXG1: Forkhead box protein G1
GPx4: Glutathione peroxidase 4
iNPCs: Neural progenitor cells differentiated from iPSCs
iNSCs: Neural stem cells differentiated from iPSCs
iPSCs: Induced pluripotent stem cells
LFS: Low-frequency stimulation
LTD: Long-term depression
LTP: Long-term potentiation
MAP2: Microtubule-associated protein 2
MEA: Microelectrode array
MERRF: Myoclonic epilepsy with ragged red fibers
MFR: Mean firing rate
MnSOD: Manganese superoxide dismutase
mtDNA: Mitochondrial genome
NGS: Next generation sequencing
NMDAR1: N-methyl-d-aspartate receptor 1
OA: Oligomycin A
OCR: Oxygen consumption rate
OXPHOS: Oxidative phosphorylation
Pax6: Paired box 6
PSD95: Postsynaptic density protein 95
ROCK: Rho-associated protein kinase
Rot: Rotenone
SDHB: Succinate dehydrogenase complex iron sulfur subunit b
SOX1: SRY-box transcription factor 1
Tuj1: Class III beta-tubulin
vGLUTs: Vesicular glutamate transporters
vGLUT1: Vesicular glutamate transporter 1
vGLUT2: Vesicular glutamate transporter 2
