# Supplementary material for: Mitochondrial impairment and synaptic dysfunction are associated with neurological defects in iPSCs-derived cortical neurons of MERRF patients
Source: J Biomed Sci. 2023 Aug 21;30:70. doi: 10.1186/s12929-023-00966-8 (PMC10441704; doi:10.1186/s12929-023-00966-8)
Supplement: Supplementary file 1 — Additional file 1: Table S1. The relationship between the heteroplasmy level of m.8344A > G mutation and its effects on the biological functions of neural cells derived from MERRF-iPSCs. [file 12929_2023_966_MOESM1_ESM.docx]

Supplementary Table 1. The relationship between the heteroplasmy level of m.8344A>G mutation and its effects on the biological functions of neural cells derived from MERRF-iPSCs.

|  | |  | MERRF iNSC MERRF neuron | | | | | |
| --- | --- | --- | --- | --- | --- | --- | --- | --- |
| Biological properties | | Regression coefficient | | R^2^ | *P*-value | Regression coefficient | R^2^ | *P*-value |
| **Respiration impairment** | |  | |  |  |  |  |  |
| ATP-coupled | -0.149 | | | 0.807 | 0.014^*^ |  |  |  |
| Maximal | -0.526 | | | 0.688 | 0.040^*^ |  |  |  |
| **ROS accumulation** | 4.177 | | | 0.567 | 0.141 | 2.191 | 0.844 | 0.027^*^ |
| **Neuron markers** |  | | |  |  |  |  |  |
| Tuj1 | |  | |  |  | -0.006 | 0.678 | 0.043^*^ |
| MAP2 | |  | |  |  | -0.006 | 0.459 | 0.138 |
| **Synaptic protein loss** | |  | |  |  |  |  |  |
| Synaptophysin | |  | |  |  | -0.011 | 0.868 | 0.021^*^ |
| vGLUT2 | |  | |  |  | -0.008 | 0.550 | 0.151 |
| **Excitatory receptor loss** | |  | |  |  |  |  |  |
| AMPARs | |  | |  |  | -0.012 | 0.841 | 0.028^*^ |
| NMDAR1 | |  | |  |  | -0.006 | 0.340 | 0.301 |
| **Neuronal function** | |  | |  |  |  |  |  |
| Spontaneous MFR | |  | |  |  | -0.029 | 0.364 | 0.204 |
| Network burst frequency | |  | |  |  | -0.001 | 0.632 | 0.107 |
| Evoked spike count | |  | |  |  | -0.543 | 0.896 | 0.014^*^ |

The values in this table were obtained by applying the Excel linear regression model analysis. *: Statistically significant.
