# Supplementary figures and images for: Mitochondrial impairment and synaptic dysfunction are associated with neurological defects in iPSCs-derived cortical neurons of MERRF patients
Source: J Biomed Sci. 2023 Aug 21;30:70. doi: 10.1186/s12929-023-00966-8 (PMC10441704; doi:10.1186/s12929-023-00966-8)

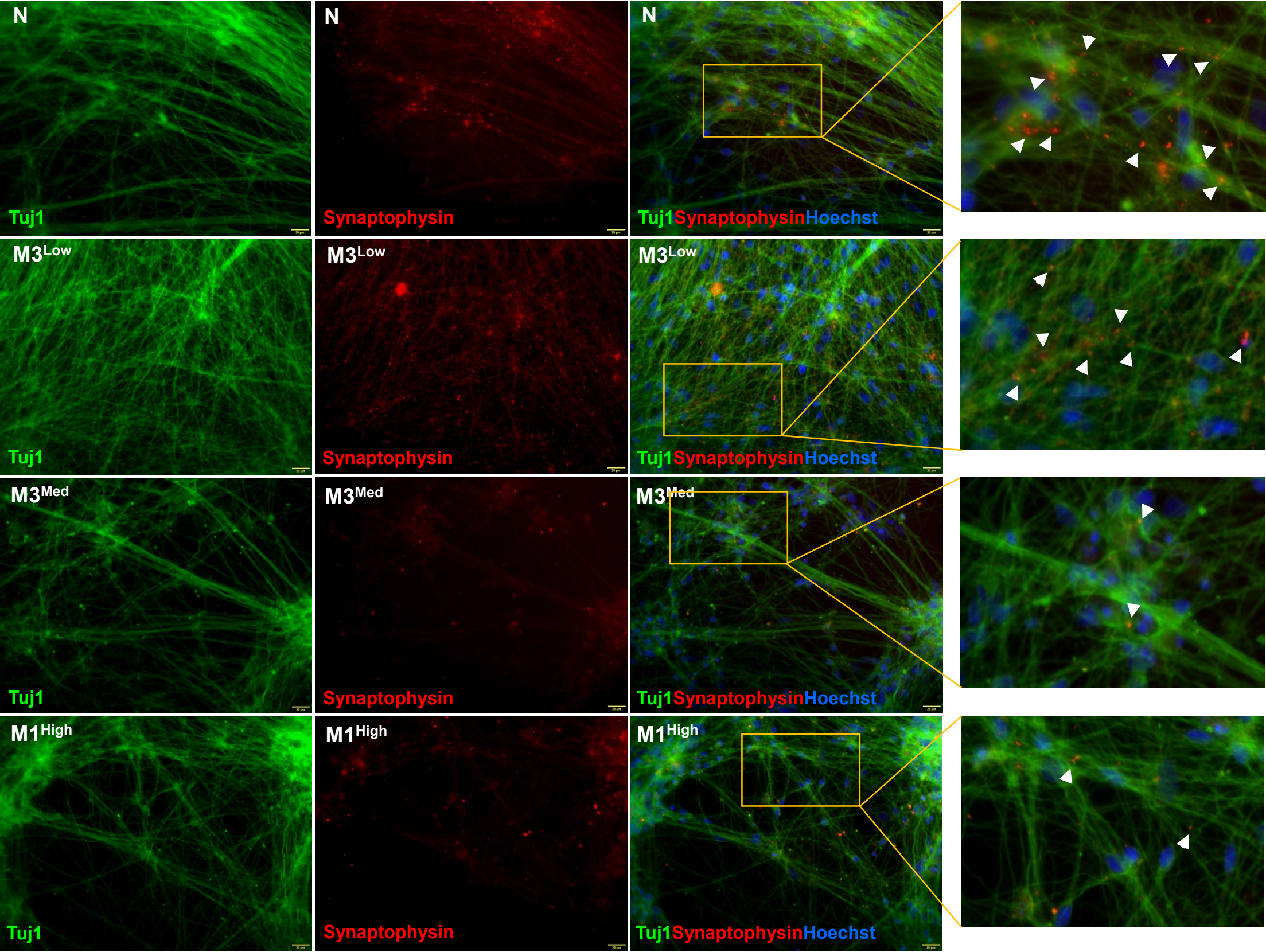

Supplement: Supplementary file 2 — Additional file 2: Figure S1. Decreased density of synaptic vesicle in the axon of neurons derived from MERRF-iPSCs harboring the m.8344A > G mutation. Distribution of synaptic vesicle in neurons 3 weeks after differentiation was analyzed by the immunofluorescence staining with antibodies against synaptophysin (red), Tuj1 (green), and Hoechst 33342 (blue). Scale bars, 20 μm. [file 12929_2023_966_MOESM2_ESM.pdf]

**A**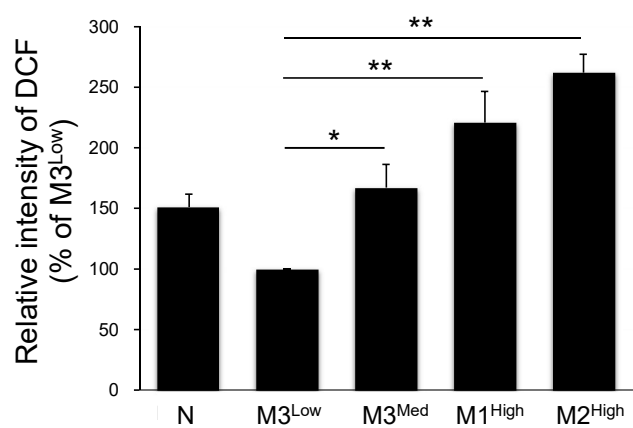**B**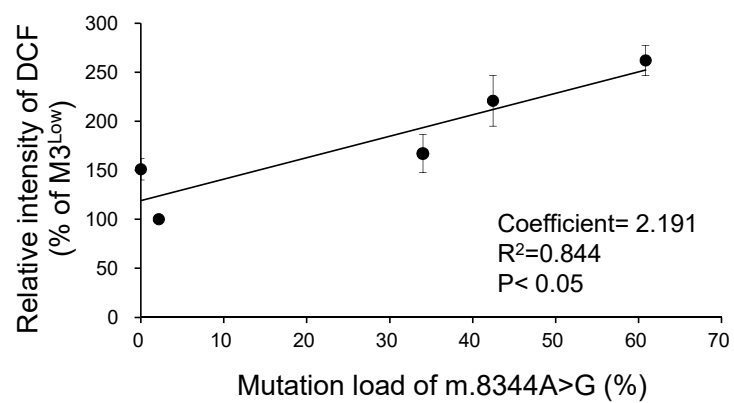

Supplement: Supplementary file 3 — Additional file 3: Figure S2. Correlation of intracellular H2O2 level and mtDNA mutation heteroplasmy in cortical neuron derived from MERRF-iPSCs harboring the m.8344A > G mutation. (A) Intracellular levels of H2O2 in normal and MERRF neurons on day 21 of neuronal differentiation were analyzed by the DCFH-dA staining of cells. The fluorescence intensity of DCF was quantified compared with that of M3Low control and displayed as the percentage change of M3Low neurons. Data are presented as mean ± SEM, n = 3. *: p < 0.05; **: p < 0.01; ***: p < 0.001. Cells were all subjected to three independent experiments except M2High, which was used to perform only two independent experiments. (B) Linear regression analysis of the m.8344A > G mutation load and the H2O2 levels in MERRF neurons. Regression of a continuous quantitative variable of mutation load with the average DCF fluorescence intensity in neurons was performed using an Excel linear regression model. (R2 = 0.844, p = 0.027, regression coefficient = 2.191). [file 12929_2023_966_MOESM3_ESM.pdf]
